# Maximum Parsimony on Phylogenetic networks

**DOI:** 10.1186/1748-7188-7-9

**Published:** 2012-05-02

**Authors:** Lavanya Kannan, Ward C Wheeler

**Affiliations:** 1 Division of Invertebrate Zoology and Richard Gilder Graduate School, American Museum of Natural History, New York, NY - 10024, USA

## Abstract

**Background:**

Phylogenetic networks are generalizations of phylogenetic trees, that are used to model evolutionary events in various contexts. Several different methods and criteria have been introduced for reconstructing phylogenetic trees. Maximum Parsimony is a character-based approach that infers a phylogenetic tree by minimizing the total number of evolutionary steps required to explain a given set of data assigned on the leaves. Exact solutions for optimizing parsimony scores on phylogenetic trees have been introduced in the past.

**Results:**

In this paper, we define the parsimony score on networks as the sum of the substitution costs along all the edges of the network; and show that certain well-known algorithms that calculate the optimum parsimony score on trees, such as Sankoff and Fitch algorithms extend naturally for networks, barring conflicting assignments at the reticulate vertices. We provide heuristics for finding the optimum parsimony scores on networks. Our algorithms can be applied for any cost matrix that may contain unequal substitution costs of transforming between different characters along different edges of the network. We analyzed this for experimental data on 10 leaves or fewer with at most 2 reticulations and found that for almost all networks, the bounds returned by the heuristics matched with the exhaustively determined optimum parsimony scores.

**Conclusion:**

The parsimony score we define here does not directly reflect the cost of the best tree in the network that displays the evolution of the character. However, when searching for the most parsimonious network that describes a collection of characters, it becomes necessary to add additional cost considerations to prefer simpler structures, such as trees over networks. The parsimony score on a network that we describe here takes into account the substitution costs along the additional edges incident on each reticulate vertex, in addition to the substitution costs along the other edges which are common to all the branching patterns introduced by the reticulate vertices. Thus the score contains an in-built cost for the number of reticulate vertices in the network, and would provide a criterion that is comparable among all networks. Although the problem of finding the parsimony score on the network is believed to be computationally hard to solve, heuristics such as the ones described here would be beneficial in our efforts to find a most parsimonious network.

## Introduction

Phylogenetic trees, or evolutionary trees, are the basic structures necessary to examine the relationships among organisms. Phylogenetic networks are generalizations of phylogenetic trees that are used to model evolutionary events when they are not only passed via vertical descent, but also by events such as horizontal exchange or recombination that cannot be modeled on a tree. Several different methods and criteria have been used to construct phylogenetic trees. The parsimony method is one such approach for inferring phylogenies, whose general idea was given in [1-3]. In this paper, our focus is on extending this approach to phylogenetic networks.

The parsimony principle states that the simplest explanation that explains the greatest number of observations is preferred over more complex explanations. Most phylogeneticists recognize that inferring genealogy rests on the principle of parsimony, that is, choosing evolutionary trees so as to minimize requirements for ad hoc hypotheses of similarity of observed characters. See [4] for a discussion on some criticisms and relevance of parsimony in phylogenetic analysis.

The cost of each character change event from a parent to child along an edge is weighted as a substitution cost of the parental state to the child state on the edge. The parsimony approach seeks a phylogenetic tree/network that, when we reconstruct the evolutionary events leading to the data on the leaves, minimizes the sum of the weights on the edges. We then face two important problems. First, we must be able to make a reconstruction of events on each vertex of the tree/network, such that the sum of the substitution costs on the edges is minimized (optimize the parsimony score on a network). Second, we must be able to search among all (or a subset of) possible phylogenetic networks for the one(s) that minimizes the parsimony score (find the network that has minimum parsimony score). This problem is NP-hard even for phylogenetic trees [5,6]; and heuristic methods have been developed to reconstruct the phylogenetic network with the given number of reticulation vertices [7,8]. In this paper, we restrict ourselves to the first of these issues, namely in establishing a parsimony criterion and to provide algorithms to achieve heuristics on finding the optimal score for any given phylogenetic network.

An often used structure to represent the evolution of sequences with reticulations is a family of trees each describing the evolution of a segment of the sequence [9,10]. In previous approaches [8,11-13], the parsimony criterion on a network has been defined as the sum total of the substitution costs on the edges of a tree (a subgraph of the network) that minimizes the parsimony score of the site. The parsimony scores for networks given in these definitions are NP-hard to compute. Moreover, a major problem with this extension is that it favors more complex evolutionary relationships by adding larger numbers of edges to trees over simpler ones that contain fewer additional edges, thus having the potential of overestimating the amount of reticulation (horizontal events) in the data. An ad hoc solution has been provided by the authors, namely to restrict blocks of contiguous sites to optimize on the same tree, rather than choosing site-specific most parsimonious tree. However, it is not clear how these blocks are chosen.

In this paper, we recall the definition of phylogenetic networks, and point out the specific class of networks for which a systematic deletion of sets of edges yields phylogenetic trees. To tackle the problem of overestimating the reticulate vertices, we define the parsimony problem simply as the sum of all mutations along all edges in the network. Thus the greater the number of edges in the network, the more the network will be penalized for having excessive number of substitutions and thus later efforts on searching the best network may identify a simpler structure with fewer reticulation events. It is also of interest to generalize the parsimony problems when the substitution costs between states are arbitrarily given. In such cases, the substitution costs along the edges of the network are stored as a cost matrix.

In the upcoming sections of the paper, we first recall the formal definition of a phylogenetic network introduced in [14], that is shown to be appropriate for various datasets. Then we will provide different parsimony criteria and some restrictions on the phylogenetic networks that offer lower complexity solutions to the previous definition. The main focus of this paper is to give a robust definition of the parsimony criterion using any given substitution cost matrix on phylogenetic networks. We also provide efficient upper and lower bounds for the optimum parsimony score on phylogenetic networks by extending the well-known Sankoff algorithm [15,16] for general cost matrix and Fitch algorithm [17] for counting the state changes along the edges of the phylogenetic trees. Our algorithm on general cost matrix works for all phylogenetic networks, thus providing a robust method to analyze any "weighted" parsimony score across the space of all phylogenetic networks. We also present an extension of the Fitch's algorithm for networks. This extension gives an upper bound on the number of state changes along the edges of the network. Additionally, for a restricted class of phylogenetic networks, defined later as phylogenetic networks with no sister reticulations, we present a method to calculate the lower bound on the number of state changes.

The parsimony criterion that we define here is simply the sum of the substitution costs along all the edges of the network. Although this total cost does not reflect the cost of the best tree in the network that displays the evolution of the site, having a overall cost of a network will be relevant while searching the space of all networks with the same number of reticulate vertices for the most parsimonious network. If needed, the tree-like evolutionary pattern of the site may later be extracted from such a parsimonious network that is found for the set of aligned DNA sequences that contains the site. This approach to search for a best network has the advantage of being much more direct than the somewhat ad hoc method that uses the criterion defined in [8]. Both our approach and the one explained in [8] would need additional cost considerations to find an appropriate number of reticulate vertices that reflects the evolutionary changes of a set of aligned DNA strings, for example. In this context, our approach has an advantage of having the score dependent on the number of reticulate edges. Later approaches to find the right number of reticulate vertices may just use some threshold on the score that proceed to consider an additional reticulate vertex if the score is above the threshold. Finding the most parsimonious network is beyond the scope of this paper, and we will only focus on efficiently computing the parsimony score defined here for a given network.

## Parsimony on phylogenetic networks

### Definition of phylogenetic networks

We follow the definition of the phylogenetic networks as given in [[14], Definition 4, page 16]. For all other graph-theoretical definitions that are not given here, we follow [18]. A rooted phylogenetic network, simply called here a *phylogenetic network*, is defined in [19] as a rooted, directed acyclic graph (DAG), whose root has indegree 0 and the leaves have outdegree 0. The vertices whose indegree is greater than 1 are called *reticulate vertices *and the edges with reticulate vertices as head vertices are called *reticulate edges*. All other edges are termed *tree edges*. The definition given in [14] takes care of the so-called "time-consistency" restraint, namely, that the tree edges take place in a positive time and the reticulate vertices have parents that can only "coexist in time". Hence, forward, we recall the formal definition of phylogenetic networks as given in [14].

Given any directed graph, we say two vertices *u *and *v cannot coexist in time *if there exists a sequence *P *= (*p*_1_, *p*_2_, ..., *p_k_*) of paths in *N *such that:

1. *p_i _*is a directed path that contains at least one tree edge, for every 1 ≤ *i *≤ *k*,

2. *u *is the tail of *p*_1_, and *v *is the head of *p_k_*, and

3. for every 1 ≤ *i *≤ *k - *1, there exists a network vertex whose two parents are the head *p_i _*and the tail of *p*_*i*+1_.

A *phylogenetic network N *is a rooted DAG obeying the following constraints:

1. Every vertex has indegree and outdegree defined by one of the four combination (0, 2), (1, 0), (1, 2), or (2, 1) - corresponding to, respectively, *root, leaves, internal tree vertices*, and *reticulate vertices*. All vertices other than reticulate vertices are called *tree vertices*.

2. If two vertices *u *and *v *cannot coexist in time, then there does not exist a network vertex *w *with edges (*u, w*) and (*v, w*).

3. Given any edge of the network, at least one of its endpoints must be a tree vertex.

Another component of this definition is that for any edge in the phylogenetic network, at least one of its endpoints (either the head or tail) is a tree vertex. Here, we will use this definition. Wherever possible, we point out whether the conditions of the definition are necessary.

**Figure 1:**
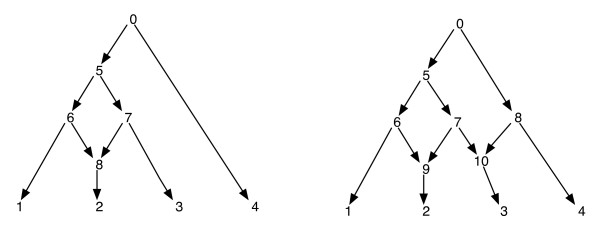
**Phylogenetic Networks**. The figure shows two phylogenetic networks; the one on the left has only a single reticulate vertex and the one on the right has two reticulate vertices that are sisters to each other. Note that removing the edges (7, 9) and (7, 10) from the network on the right does not result in a tree where vertex 7 is a leaf. This shows that removing one incoming edge each per reticulate vertex does not necessarily produce a tree with the same leaf set as the network. The post-ordering and the pre-ordering of vertices of the network on the left are 1, 2, 8, 6, 3, 7, 5, 4, 0 and 0, 5, 6, 1, 8, 2, 7, 3, 4 respectively and for the network on the right, they are 1, 2, 9, 6, 3, 10, 7, 5, 4, 8, 0 and 0, 5, 6, 1, 9, 2, 7, 10, 3, 8, 4 respectively.

Phylogenetic networks can naїvely be thought of as a network that contain as subgraphs, the trees that explain the evolutionary histories of different segments of the input terminal sequences. Given a phylogenetic network, deleting one of each edge incident to a reticulate vertex does not guarantee a resulting phylogenetic tree with the same set of leaves as that of the network. This is an undesirable property, especially if the parsimony criterion is defined by finding a phylogenetic tree inside the network that is most parsimonious for the given site, as defined in [8,11-13]. In order to avoid this problem, it is necessary to assume that no internal vertex has two reticulate children. We call this class of phylogenetic networks as a *phylogenetic network with no sister reticulations*. See Figure 1 for some examples of phylogenetic networks.

Before we proceed to the definition of the parsimony problems, the following is a useful observation. For a phylogenetic network *N *with no sister reticulations, and having *r *reticulate vertices and with leaf set *X*, we denote T(N) as the set of all trees contained in *N*. Each such tree is obtained by following two steps: (1) for each reticulate vertex, remove one of the incoming edges, and then (2) for every vertex *v *of indegree and outdegree 1, whose parent is *u *and child is *w*, contract the edges (*u, v*) and (*v, w*) into a single edge (*u, w*). The condition that each edge in *N *has a tree vertex as an endpoint and that each tree vertex has at least one tree vertex as a child, ensures that the set of leaves of the resulting tree is the same as that of the network. Hence the set T(N) contains exactly 2^*r *^phylogenetic trees whose leaf set is exactly *X*.

### Maximum Parsimony

We refer the readers to [2,3] for a general description of the idea of parsimony and to the discussion of various parsimony algorithms. It has been pointed out in [9] that the parsimony method for trees can be extended to phylogenetic networks. In a series of papers [8,11,12], one such parsimony criterion is defined by finding a tree in the network that has the best parsimonious score, and efficient algorithms to optimize this criterion on a given phylogenetic network have been devised. Although these algorithms are shown to perform well in practice, they can perform correctly only for phylogenetic networks with no sister reticulations, since it is straightforward to search for an optimal tree in these restricted class of networks. In this section, we state an alternate version of the parsimony problem and in the following sections provide some heuristic solutions for optimizing the score on any phylogenetic network.

Let [*n*] = {1, 2, ..., *n*} denote the set of leaf labels of a given phylogenetic network *N*. A function *λ: *[*n*] **→ **{0, 1, ...,|Σ| - 1} is called a *state assignment function *over the alphabet Σ (a non-empty set) for *N*. We say that a function $\hat{\lambda }:V\left(N\right)\to \left\{0,\;1,\;\ldots \;,|\Sigma |-1\right\}$ is an *extension of λ on N *if it agrees with *λ *on the leaves of *N*. For a vertex *v *in *N*, we call the $\hat{\lambda }\left(v\right)$ as an *assignment *of $\hat{\lambda }$ on *v*. A fully assigned network is a network in which all the vertices have labels from {0, 1, ..., |Σ| - 1}. Let *C *be a cost matrix whose *ij*^th ^entry *c_ij _*is the cost of transforming from state *i *to state *j *along any edge in *N*. If *e *= (*u, v*) is an edge in *N*, where *u *is the parent of *v*, we denote $w_{e}\hat{\left(\lambda \right)}=c_{ij}$, where $i=\hat{\lambda }\left(u\right)$ and $j=\hat{\lambda }\left(v\right)$. For a graph *G*, we let *E*(*G*) denote the edge set of *G*. Then the parsimony problem is defined as follows.

*Input: *A phylogenetic network *N *with leaf labels [*n*] and a state assignment function *λ *over the alphabet Σ for *N*.

*Parsimony criterion: *For an extension $\hat{\lambda }$ of *λ*, let

$$P_{1}\left(\hat{\lambda }\right)=\underset{T\in T\left(N\right)}{\text{min}}\underset{e\in E\left(T\right)}{\sum }w_{e}\left(\hat{\lambda }\right),$$

and

$$P_{2}\left(\hat{\lambda }\right)=\underset{e\in E\left(N\right)}{\sum }w_{e}\left(\hat{\lambda }\right).$$

*Output: *Given *P *∈ {*P*_1_, *P*_2_}, find $\hat{\lambda }$ that minimizes $P\left(\hat{\lambda }\right)$.

We note that $P_{1}\left(\hat{\lambda }\right)$ is introduced in [8] and $P_{2}\left(\hat{\lambda }\right)$ is the definition we will use in this paper. A more general approach is to minimize $Q\left(\hat{\lambda }\right)=\sum_{e\in E\left(N\right)}d_{e}\left(w_{e}\left(\hat{\lambda }\right)\right)$, where *d_e _*is a non-negative weight function on the edges of *N*. For the purposes of this paper, we restrict ourselves to *P *= *P*_2_, although the first of our approaches, the dynamic programming solution also holds for *P *= *Q*.

### Parsimony algorithms on networks

#### Traversing a phylogenetic network

In a network, *vertex traversal *refers to the process of visiting each vertex, exactly once, in a systematic way. Such traversals are classified by the order in which the vertices are visited. We need two types of network vertex traversals to describe our algorithms. These are well-known for phylogenetic trees, and we present them here for phylogenetic networks. The algorithms for the traversals given below start from any given vertex *v *in the network. In this paper, we will always perform the traversals from the root vertex of the network.

Pre-order traversal of a phylogenetic network from a vertex v

1. Visit the vertex *v*.

2. Recursively perform pre-order traversal from each child that has not yet been visited.

Post-order traversal of a phylogenetic network from a vertex v

1. Recursively perform post-order traversal from each child that has not yet been visited.

2. Visit the vertex *v*.

Since a phylogenetic network is a DAG, such traversals will visit all the vertices of the network exactly once. (Refer to [18] for more details on existence on such traversals on DAGs). For the purposes of this paper, we assume that the vertices of a network are uniquely labelled by integers. Note that the leaves are already labelled from the set [*n*]; and so we use other integers for other vertices. Whenever the child vertices of *v *are extracted, they are also arranged in increasing order of their integer labelings and the pre- and post-order traversals are performed in this order. This will ensure the following: if vertices *v *and *v' *are such that there is no directed path between them, then the vertex *v *is traversed prior to vertex *v' *in the pre-order if and only if the vertex *v *is traversed prior to the vertex *v' *in the post-order. See the Figure 1 for some examples. With this property, we notice that the pre- and post-order traversals from the root of a phylogenetic network each trace the same spanning tree, which we call here the *traversal tree*.

#### Dynamic Programming solution

Dynamic programming is used to provide efficient solutions for finding the exact parsimony score when the network is a phylogenetic tree [15,16]. In this section, we show that the same approach can be generalized to phylogenetic networks. Sankoff's algorithm on a tree traverses the vertices of the tree via post-order while computing the minimum costs of each state at each vertex from the leaves to the root, and then chooses the best assignments on each vertex by backtracking from the root to the leaves by traversing the tree vertices via pre-order. Both the phases are presented for networks in Algorithms 1 and 2 respectively. We describe them briefly below. It can be noted that if the network is a tree, then our algorithms match with the pre-order and post-order phases of Sankoff's method for trees.

Given a phylogenetic network *N*, with leaf vertices labeled [*n*] and with state assignment function *λ *over the alphabet Σ, assign to each vertex *v *∈ *V *a quantity *S_v _*(*i*) for each *i *∈ Σ. In phylogenetic trees, *S_v _*(*i*) denotes the minimum sum of costs of all the events from the vertex *v *to all the leaves that are reachable from *v*, given that *v *is assigned state *i *and all the descendant vertices from *v *are each assigned a state. In networks, there is no simple way to compute such a quantity. Instead, we allow *S_v _*(*i*) to be a lower-bound of the above exact score and it is calculated during the post-order traversal phase.

*Post-order traversal phase: *If *v *is a leaf of *N*, then *S_v _*(*i*) is assigned 0 if the observed state is state *i*, and infinite otherwise. Now all we need is a recursion relationship to calculate *S_v _*(*i*) for rest of the vertices. For each child *w *of *v*, we say *w *satisfies the *post-order traversal condition with respect to v*, or simply *traversal condition with respect to v *in view of the observation in the beginning of this section, if the following hold:

(i) The vertex *w *is a reticulate vertex and

(ii) if *v' *is the parent of *w *other than *v*, then the vertex *v *must be traversed prior to *v' *in the post-order traversal of *N*.

We now define recursively for each edge (*v, w*),

$$s_{\left(v,w\right)}\left(i\right)=\left\{\begin{array}{cc} {\text{min}}_{j}\left[c_{ij}+S_{w}\left(j\right)\right] & \text{if }w\text{ satisfies the traversal condition with respect to }v\text{;}\\ {\text{min}}_{j}c_{ij} & \text{otherwise}\text{.} \end{array}\right)$$

For a phylogenetic tree, *s*_(*v, w*) _(*i*) always assumes the first of these quantities, and it thus gives the sum of the substitution costs along the edges of the tree that lie below the vertex *v*, provided the vertex *v *is assigned the state *i*. For phylogenetic networks, in order to account for the substitution costs along the edges that lie below a reticulate vertex *w *just a single time when vertex *v *is assigned the state *i*, we let the 'parent' *v *of *w *in the traversal tree account for all the substitution costs along all the edges that lie below *v*. On the other hand, if *v *is not a parent of *w *in the traversal tree, *s*_(*v, w*) _(*i*) simply denotes the substitution cost from state *i *at vertex *v *to another state at *w *that is least expensive.

We then define

(1) $$S_{v}\left(i\right)=\underset{w}{\sum }s_{\left(v,w\right)}\left(i\right),$$

where the sum runs for all child(ren) vertex(s) *w *of *v*. As mentioned before, in phylogenetic trees, *S_v _*(*i*) denotes the minimum possible sum of substitution costs along all the edges from the vertex *v *to all the leaves that are reachable from *v*, given that *v *is assigned state *i *and all the vertices reachable from *v *are each assigned a state.

In phylogenetic networks, while calculating *s*_(*v, w*) _(*i*) where *w *is a reticulate vertex such that (*v, w*) is not an edge in the traversal tree, there is no prior knowledge of the state that will be later assigned at the reticulate vertex *w*. Thus *s*_(*v, w*) _(*i*) can only be a lower bound of the edges of the network that lie below the vertex *v*, if the vertex *v *is assigned the state *i*. The reasoning for this is that *s*_(*v, w*) _(*i*) is the substitution cost from state *i *at vertex *v *to another state at *w *that is least expensive, instead of the substitution cost from state *i *at *v *to the state at *w *that will be later assigned. Since the definition of *S_v _*(*i*) depends on the definition of *s*_(*v, w*) _(*i*), and they are defined recursively, we observe the following: *S_v _*(*i*) is a lower bound on the sum of substitution costs along the edges of the network that are reachable from the vertex *v*, provided that *v *is assigned state *i *and all the descendant tree vertices are assigned a unique state, and the reticulate vertices are assigned two states that are not necessarily the same. The assigned states of the reticulate vertex contributes to a *conflict *if the states are not the same. Let us suppose that state *i *is assigned to the root vertex *r*, and all tree vertices are assigned a unique state, while the reticulate vertices are assigned two states. Then the cost *S_r _*(*i*) denotes the minimum possible sum of substitution costs along all the edges of a traversal tree with one of states assigned for reticulate vertices, plus the sum of the substitution costs along the remaining reticulate edges with the alternate assignment state at the reticulate vertices. Since we seek an assignment on the vertices of the network with no conflicts in the reticulate vertices, *S_r _*(*i*) is a lower bound on the cost of such assignment where the root vertex is assigned *i *and all vertices are assigned with a unique assignment.

During this phase, we also store the states

(2) $$t_{\left(v,w\right)}\left(i\right)=\left\{\begin{array}{cc} arg{\text{min}}_{j}\left[c_{ij}+S_{w}\left(j\right)\right] & \text{if }w\text{ satisfies the traversal condition with respect to }v\text{;}\\ arg{\text{min}}_{j}c_{ij} & \text{otherwise}\text{.} \end{array}\right)$$

to be able to backtrack the state of *w *that achieves the quantity *s*_(*v, w*) _(*i*) during the pre-order phase. See Algorithm 1.

*Pre-order traversal phase: *We first choose the minimum

$$S=\underset{i}{\text{min}}S_{r}\left(i\right)$$

where *r *is the root vertex and assign the state that attains the minimum at the root vertex, *i.e*., let $\hat{\lambda }\left(r\right)=i_{r}$ such that *S_r _*(*i_r_*) = *S*. For any other vertex *w *that is not a reticulate vertex, whose parent *v *is already assigned with a state *i*, we assign the state *t*_(*v, w*) _(*i*). For a reticulate vertex *w *whose parent vertices are *v *and *v'*, let us suppose that *v *and *v' *are assigned states *i *and *i' *respectively when traversing by the pre-order. The possible states *j *= *t*_(*v, w*) _(*i*) and *j' *= *t*_(*v', w*) _(*i'*) of *w *that achieve *s*_(*v, w*) _(*i*) and *s*_(*v', w*) _(*i'*) respectively, need not be the same. In other words, it is possible that *j *≠ *j'*. In this case, we have a *conflict *on the reticulate vertex *w*. Thus, the dynamic programming technique fails to give an extension for *λ *whose parsimony score is *S*. In this case, we simply choose between *j *and *j' *for *λ*(*w*) according to which of the vertices among *v *and *v' *is traversed first in the pre-order. Thus, if the vertex *w *satisfies the traversal condition with respect to *v *we have $\hat{\lambda }\left(w\right)=j$.

**Figure 2:**
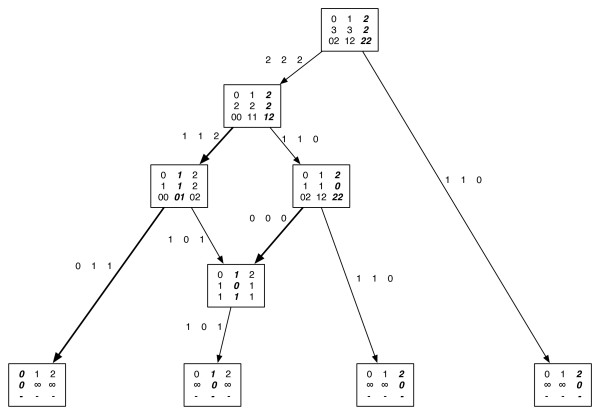
**Dynamic programming solution**. The dynamic programming solution applied to a phylogenetic network. The states are 0, 1 and 2. The cost matrix used has all 1s except the diagonal elements which are all 0s. The tables shown on each vertex *v *are the costs, *S_v _*(*i*) (second row) of each state, *i *(first row) that are computed during the post-order traversal. Also shown at the vertices are the states of the child *w*, namely the *t*_(*v, w*) _(*i*) (third row) that correspond to the costs in the second row; when there are two children for a vertex, the entries in the third row are represented as a pair of states of the left child and the right child respectively. Each edge (*v, w*) is labelled with *s*_(*v, w*) _(*i*) for each state *i*. During the pre-order traversal, the states for each vertex are selected (shown in bold). The cost of 2 highlighted in bold at the root vertex gives a lower bound, *S*. The state assigned at each vertex is highlighted in bold. The algorithm finds a total of three substitutions (highlighted by bold edges). This is because the states assigned at the parent vertices of the reticulate vertex give conflicting assignments of 1 and 2 respectively, of which state 1 is assigned at the reticulate vertex. Thus with an extra cost of 1, we get the score of 3 (an upper bound of the optimal score) as the parsimony score corresponding to the assignment shown. Note that the optimum parsimony score on the network is 2 (equal to the lower bound), which can be found by exhaustive search and can be realized by changing the assignment from 1 to 2 for the left parent of the reticulate vertex and from 1 to 2 for the reticulate vertex. Thus the lower bound matches with the optimal score, although the assignment corresponding to the lower bound is not conflict-free and not the same as the assignment corresponding to the optimum.

After completing the pre-order phase, we can get the score corresponding to the extension $\hat{\lambda }$ by first setting *S' *= *S *and updating *S' *at each reticulate vertex *w *as follows: The upper bound score *S' *is updated corresponding to the assignment *j *at vertex *w *as *S' -c_i' j' _*+*c_i' j_*. See Algorithm 2. Figure 2 shows an example of how the algorithm runs on a network. Since *S_r _*(*i*) is a lower bound on the optimum assignment where the root vertex is assigned *i *and all vertices are assigned with a unique assignment, and since *S *= min_*i *_*S*_*r *_(*i*), we conclude that *S *is a lower bound of the optimum we seek to find. See Lemma 1 for a formal proof.

**Lemma 1**. *The quantity S is a lower bound of the optimum parsimony score on the network N*.

*Proof*. By the construction of *S*, we have

(3) $$S=\underset{\left(v,w\right)\in E\left(N\right):w\;\text{is a tree vertex}}{\sum }c_{\hat{\lambda }\left(v\right),\hat{\lambda }\left(w\right)}+\underset{\left(v,w\right),\;\left(v^{\prime },w\right)\in E\left(N\right)}{\sum }\left[c_{\hat{\lambda }\left(v\right),\hat{\lambda }\left(w\right)}+c_{\hat{\lambda }\left(v^{\prime }\right),t\left(v^{\prime },w\right)\left(\hat{\lambda }\left(v^{\prime }\right)\right)}\right],$$

where the second summand is for the reticulate vertex *w *with parents are *v *and *v'*, such that *v *satisfies the traversal condition w.r.t. *w*. Thus the cost $c_{\hat{\lambda }\left(v\right),\hat{\lambda }\left(w\right)}$ is the substitution cost from the assigned state $\hat{\lambda }\left(v\right)$ at *v *to the state $\hat{\lambda }\left(w\right)$ at *w*. On the other hand, the cost $c_{\hat{\lambda }\left(v^{\prime }\right),t_{\left(v^{\prime },w\right)}\left(\hat{\lambda }\left(v^{\prime }\right)\right)}$ is the substitution cost from the assigned state $\hat{\lambda }\left(v\right)$ at *v *to the state $t_{\left(v^{\prime },w\right)}\left(\hat{\lambda }\left(v^{\prime }\right)\right)$ at *w*. Note that the state $t_{\left(v^{\prime },w\right)}\left(\hat{\lambda }\left(v^{\prime }\right)\right)$ is not necessarily same as the state $\hat{\lambda }\left(w\right)$, and *S *is the minimum among all assignments that may result in conflicts at the reticulate vertices.

Suppose $\hat{S}$ is the optimum parsimony score on *N *with the function *μ: V *(*N*) → {0, 1, ..., |Σ| - 1} as the extension of *λ *we have

(4) $$\hat{S}=\underset{\left(v,w\right)\in E\left(N\right):w\;\text{is a tree vertex}}{\sum }c_{\mu \left(v\right),\;\mu \left(w\right)}+\underset{\left(v,w\right),\;\left(v^{\prime },w\right)\in E\left(N\right)}{\sum }\left[c_{\mu \left(v\right),\mu \left(w\right)}+c_{\mu \left(v^{\prime }\right),\mu \left(w\right)}\right],$$

where in the second summand *w *is a reticulate vertex with parents *v *and *v'*. Since *μ *is a conflict-free assignment that is contained in the set of all assignments among whose costs *S *is the minimum (compare equation (3) and (4)) we have $S\leq \hat{S}$. □

Now for the complexity of the algorithm. Suppose the network *N *has *n *leaves and *r *reticulate vertices. Then the number of vertices in *N *is 2(*n *+ *r*) -1. At each vertex *v *and for each state *i*, the quantity *S *can be computed in *O*(*k*^2^) time, where *k *= |Σ|. The pre-order traversal step involves finding *S *in *O*(*k*) complexity and assigning the best states for each vertex. Also, fixing conflicting reticulate vertex states takes *O*(*r*) time. Thus the complexity of the algorithm (presented here) to find a lower and an upper bound is *O*((*n *+ *r*)*k*^2^). An alternate upper bound can be obtained in *O*(*nk*^2^) by simply assigning during the post-order traversal phase, for each reticulate vertex the state that occurs the maximum number of times at the leaves reachable from the respective reticulate vertex; and proceeding via finding *S_v_*(*i*) for the remaining vertices. The exact optimum can also be obtained by restricting the possible states to a single state for each reticulate vertex, by running the dynamic programming algorithm for each of the *k^r ^*combinations of states for the reticulate vertices, and choosing the minimum among all of them. The time-complexity of this process is *O*(*nk*^*r*+2^).

**Algorithm 1 **Post-order traversal phase: Calculate the cost of each state at each vertex

1: Input: Network *N *and the observed states from Σ at the leaves of *N, i.e*., a state assignment function *λ *over the alphabet Σ for *N*.

2: For each leaf *v*, let *S_v _*(*i*) = 0 if *λ*(*v*) = *i *and ∞ otherwise.

3: Repeat in post-order for each in internal vertex (root, internal tree vertex or reticulate vertex) *v *in *N*: For each state *i*, compute *S_v _*(*i*) given in (1) and *t*_(*v, w*) _(*i*) for each child *w *of *v*, given in (2).

4: Output: {(*S_v_*(*i*), [*t*_(*v, w*)_(*i*): *w *is a child of *v*]): *v *∈ *V *(*N*), *i *∈ Σ}.

#### Minimizing the number of mutations on a phylogenetic network

**Figure 3:**
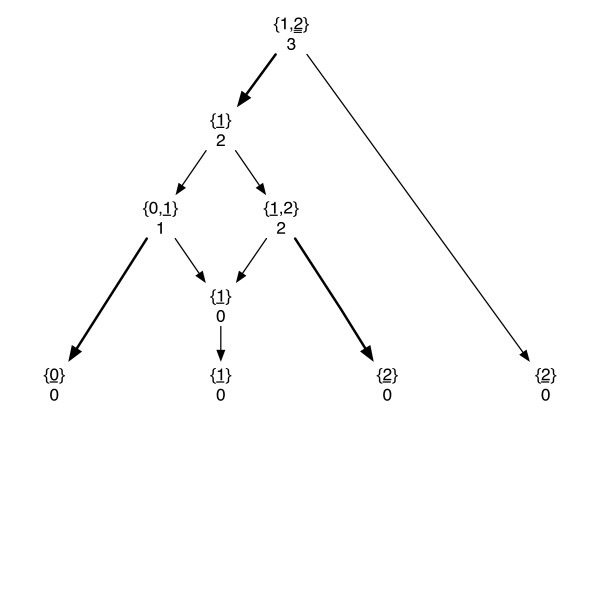
**Fitch-type solution**. The Fitch-type solution applied to the same phylogenetic network and the leaf data in Figure 2. Each vertex is assigned a set of all possible states, along with a score when the network vertices are traversed in post-order. The score at the root gives a upper bound for the optimal score. The state assignments are given during the pre-order traversal phase and the number of substitutions matches with the score at the root.

The Fitch algorithm [17] counts the number of changes in a bifurcating phylogenetic tree for any character set, where the states can change from any state to any other state. Thus, the cost matrix is such that its diagonal elements are all zeros and the off-diagonal elements are all ones. In this section, we show how Fitch's algorithm extends to finding upper and lower bounds for the number of evolutionary changes in a given phylogenetic network. First, we show that the Fitch algorithm can be extended to give an upper bound for the optimum parsimony score. As before, the post-order and the pre-order traversal phases are given in Algorithms 3 and 4 below. See Figure 3 for an example run of the algorithm.

**Algorithm 2 **Pre-order traversal phase: Calculate lower and upper bounds of the optimum and the corresponding assignment of the upper bound

1: Input: {(*S_v _*(*i*), [*t*_(*v, w*) _(*i*): *w *is a child of *v*]): *v *∈ *V *(*N*), *i *∈ Σ}.

2: Let *S *= min_*i *_*S*_*r *_(*i*), where *r *is the root vertex and let $\hat{\lambda }\left(r\right)=arg{\text{min}}_{i}S_{r}\left(i\right)$.

3: Let *S' *= *S*

4: For each vertex *w *in pre-order whose parent vertex *v *immediately preceeds *w *in the pre-order, let $\hat{\lambda }\left(\omega \right)=\;t_{\left(v,\;w\right)}\left(i\right),$ where $i=\hat{\lambda }\left(v\right)$.

5: Visit each reticulate vertex *w *with parents *v *and *v' *such that *w *satisfies the traversal condition with respect to *v*, with $i=\hat{\lambda }\left(v\right)$, $i^{\prime }=\hat{\lambda }\left(v^{\prime }\right)$, $j^{\prime }=t_{\left(v^{\prime },w\right)}\left(i\right)$ and update *S' *as follows:

$$S^{\prime }\leftarrow S^{\prime }-c_{i^{\prime }\;j^{\prime }}+c_{i^{\prime }\;j}.$$

6: Output: (Lower bound, Upper bound) = (*S, S'*); extension corresponding to the upper bound score $S^{\prime }:\hat{\lambda }$.

**Algorithm 3 **Post-order traversal phase: Calculate the optimum

1: Input: Phylogenetic network *N *and a state assignment function *λ *over the alphabet Σ for *N*.

2: For every leaf *v *of *N*, we are given *A*(*v*) = {*λ*(*v*)}, a singleton set containing the observed state at the leaf.

3: Set *UB *= 0.

4: Recurse using post-order: For a vertex *v *of *T *with children *w*_1 _and *w*_2_, let

$$A\left(v\right)=\left\{\begin{array}{cc} A\left(w_{1}\right)\cap A\left(w_{2}\right) & \text{if }A\left(w_{1}\right)\cap A\left(w_{2}\right)\neq 0̸;\\ A\left(w_{1}\right)\cup A\left(w_{2}\right) & \text{otherwise}\text{.} \end{array}\right)$$

and

$$UB\leftarrow \left\{\begin{array}{cc} UB & \text{if}\;A\left(v_{1}\right)\cap A\left(v_{2}\right)\neq 0̸;\\ UB+1 & \text{otherwise}\text{.} \end{array}\right)$$

If the vertex *v *has a single child *w*, then

A(v)=A(w),

and

UB←UB.

5: ({*A*(*v*): *v *∈ *V *(*N*)}, *UB*)

Since the pre-order traversal phase gives a conflict-free assignment on the vertices, *UB *is an upper bound. This is a special case of the dynamic algorithm presented for general cost-matrix. Suppose we restrict *N *to be a phylogenetic network with no sister reticulations, then any Fitch solution on any tree *T *in T(N) forms a lower bound for the optimal score on networks; and adding the cost on edges not in *T *gives an upper bound for the optimal score. Thus, it is possible to calculate our lower bound for counting the number of character changes only for phylogenetic networks with no sister reticulations, where it is straightforward to find a tree in T(N).

**Algorithm 4 **Pre-order traversal phase: Assigning the states

1: Input: Phylogenetic tree *N *and ({*A*(*v*): *v *∈ *V *(*N*)}, *UB*).

2: For every vertex *v *in the tree that is not already assigned, the algorithm computes $\hat{\lambda }\left(v\right)$ as follows: For the root *r*, $\hat{\lambda }\left(r\right)=\sigma$, where *σ *is an arbitrary element of *A*(*r*). Assign recursively via pre-order: For a vertex *v *whose parent *u *is assigned,

$$\hat{\lambda }\left(v\right)=\left\{\begin{array}{cc} \hat{\lambda }\left(u\right) & \text{if }\hat{\lambda }\left(u\right)\in A\left(v\right);\\ \sigma \in A\left(v\right) & \text{otherwise}\text{.} \end{array}\right)$$

3: Fixing the score: for each reticulate vertex *v*, if *u' *is not the parent in pre-order, and if $\hat{\lambda }\left(u^{\prime }\right)\in A\left(v\right)$, but $\hat{\lambda }\left(u^{\prime }\right)\neq \hat{\lambda }\left(v\right)$, then increment *UB *by 1.

4: Output: *UB *and extension function $\hat{\lambda }$ of *λ*.

## Discussion and conclusion

In the maximum parsimony problem, there are known character-states for a set of taxa (of the species) or Operational Taxonomic Units (OTUs). The problem is to find an order of branching and an ancestral configuration of character-states requiring the minimum number of character-state changes to account for the descent of the OTUs. Short of searching all possible networks, the problem is still in the early stage of being addressed. A more modest goal is to find maximum parsimony ancestral character-states for which both the current character-states and the network are known.

In this paper, we extend the parsimony score defined on phylogenetic trees to phylogenetic networks. This score is defined as the sum of all the substitution costs along all edges of the network. This approach provides an estimate on the amounts of substitutions along all edges, and hence later efforts to find networks with optimal score will fetch networks with fewer reticulations. Although the complexity of finding the exact score on a given network is unknown, we suspect that the problem will also be NP-hard as with the definition of the problem via previously defined criterion. We extended Sankoff and Fitch algorithms that are well-known for trees to heuristic algorithms on networks that compute upper and lower bounds for then optimal parsimony score. Sankoff's algorithm works for any general substitution cost matrix, and our extension also provides a robust method to calculate heuristic bounds for the optimal score on networks with non-homogeneous substitution costs.

We ran our algorithm for networks with fewer than 10 leaves with at most 2 reticulation events and found that for all these networks, the bounds matched with the exact optimum, which we were able to compute using our exact algorithm. Future efforts in this area of research will involve tightening these bounds for general phylogenetic networks. This will enable us to proceed to the next step of the parsimony problem, namely to find the networks with optimum parsimony score.

## Competing interests

The authors declare that they have no competing interests.

## Authors' contributions

WW conceived the study, and participated in its design and coordination, LK implemented the algorithms in OCAML. LK wrote the paper and WW proofread all versions of the manuscript during its preparation. All authors read and approved the final manuscript.
